# Supplementary material for: Information-based centralization of locomotion in animals and robots
Source: Nat Commun. 2019 Aug 13;10:3655. doi: 10.1038/s41467-019-11613-y (PMC6692360; doi:10.1038/s41467-019-11613-y)
Supplement: Supplementary file 1 — Supplementary Information [file 41467_2019_11613_MOESM1_ESM.pdf]

Supplementary Information for

Information-Based Centralization of Locomotion in Animals and Robots

Neveln et al.

Corresponding Author Izaak Neveln.
E-mail: ineveln2@gmail.com

A. Supplementary Note 1: From Experiment to Centralization.

Here we give the five general steps followed in this article to get from running the experiment to measuring centralization and co-information. For more details on these steps see the following notes and the methods in the main text.

1. Collect Signals From the System. Measuring centralization requires a large sampling of the specific behavior or task. All that is required is the continuous measurement of the control signal, the local state, and the global state of the system. For the cockroach, this required EMG electrode recording synced with high speed video, whereas the robot internally logged torque estimates and motor positions from onboard sensors. The sampling of these time series should be stationary, i.e. the statistics of the signals should be constant over time. Therefore, the behavior should not be changing so much that the overall distributions of these signals change.

2. Organize Signals Into Observations. To build a distribution of the measured signals, we must organize the data into independent observations. For the case of baseline running, this means organizing the trajectories into strides and normalizing the data to the stride period. In the coupled-oscillator model, each observation was a fixed window of time after a perturbation. Perturbation studies could also be applied in animals or robots, though in this work we consider each step as a random perturbation rather than a using prescribed perturbations in order to quickly collect the number of strides needed.

3. Parameterization or Dimensionality Reduction. Often the raw signals are continuous and therefore high dimensional if considered as a time series of variables over discrete units of time, like strides. However, these signals can usually be represented in a lower dimensional space with little reduction in the variance of the signals. In the case of EMG activity, the spikes are discrete events and can be parameterized by the number (count) and timing of those events. Other trajectories can be reduced in dimension in other ways as described in the *Dimensionality Reduction* section. This process is essential for the calculation of mutual information as the number of observations needed for a stable estimate increases dramatically with the dimension of the signals.

4. Mutual Information Estimation. Three mutual information values are estimated between the distribution of the control signal parameters and three distributions of the state parameters: the local state distribution, the global state distribution, and the joint distribution of both states. Details on the theory underlying these three measures as well as how the estimate is achieved is given in subsequent sections.

5. Estimation Validation. As the mutual information estimates could be biased due to a poor sampling of the underlying probability distribution, we check the stability of the estimates to subsampling and the method of dimensionality reduction. We detail this procedure in a subsequent section.

B. Supplementary Note 2: Background on Information Theory.

Entropy and Mutual Information. The discrete Shannon entropy (H) of a signal, given by the equation

$$H(S) = -\sum_i p(s_i) \log p(s_i), \qquad [1]$$

quantifies the amount of information present in the signal, where s_i is each possible state the signal S can take and p is the probability distribution of the states (1). When the base of the logarithm is two, the unit of entropy is the bit, where the number of bits represents the expected number of yes or no questions to determine the state of the signal. Entropy can be similarly defined for joint distributions as $H(S_1; S_2)$, i.e. the entropy of the set of random variables S_1 and S_2, and conditional distributions as $H(S_1|S_2)$, i.e the entropy in S_1 given that S_2 is known.

$$H(S_1; S_2) \leq H(S_1) + H(S_2) \qquad [2]$$

with equality only when the two signals are independent. The level of interdependence between two signals is quantified by the mutual information I, which is the difference from equality in Eq. 2 given by the equation

$$I(S_1; S_2) = H(S_1) + H(S_2) - H(S_1, S_2). \qquad [3]$$

Mutual information can also be written as

$$I = H(S_1) - H(S_1|S_2) = H(S_2) - H(S_2|S_1). \qquad [4]$$

Therefore, the mutual information measures the decrease in entropy in one signal when the state of the other signal is known. These overlapping entropies for our chosen set of signals (hereafter labeled C for the set of possible c_i control states, L for the set of local states, and G for the set of global states) are graphically presented in Supplementary Figure 1 and Fig. 1B. Estimation of mutual information of continuous variables can have error or bias due to limited sampling (2). We use a bin-less nearest neighbor estimator of I which handles these issues well (2), as described in the next section.

Trivariate Information Decomposition. We are specifically interested in understanding the composition of the total mutual information I_{TOT} shared between a particular C and both states L and G. I_{TOT} is represented in Supplementary Figure 1 by area encompassed by the dotted black line. An intuitive decomposition of I_{TOT} into four separate positive values is given by

$$I_{TOT} = I_{UL} + I_{UG} + I_R + I_{SYN}, \tag{5}$$

and is shown in Supplementary Figure 2. I_{UL} and I_{UG} represent information shared uniquely between the control signal and the local and global states respectively, I_R is redundant information shared when either of local or global states are known, and I_{SYN} is synergistic information shared only when both states are known (3, 4). The axioms that allow for such a decomposition are debated, and estimating these quantities becomes challenging due to the need to optimize the estimate according to the chosen axiom (5). We avoid estimating these quantities directly, as we can compute differences of these quantities that are useful measures of the systems we study from our estimates of local MI I_L, global MI I_G, and total MI I_{TOT} as shown in Supplementary Figure 2.

The entropy diagram in Supplementary Figure 1 and the schematics of the decomposition in Supplementary Figure 2 help build intuition about how the different mutual information quantities contain different constituent parts of the total mutual information between the control signal and the joint distribution of local and global states. Local mutual information outlined by the dashed red line is the red and gray areas together in Supplementary Figure 1 and is given by

$$I_L = I_{UL} + I_R. \tag{6}$$

This is the mutual information between C and L when G is not known. When G is known, then the red area in Supplementary Figure 1 is given by

$$I(C;L|G) = I_{UL} + I_{SYN} \tag{7}$$

and does not include I_R. As there is no I_{SYN} in I_L, the grey area must contain negative I_{SYN} to cancel out the positive I_{SYN} in the red area along with positive I_R.

As a quick side note about synergistic information, this term could be conflated with the idea of muscle synergies (6) which says that muscles often act together in various coordinated groups. Activity in one muscle would then be considered to have redundant information with activity in another muscle with regards to the output state. Synergistic information here is when the muscle activity shares more with both local and global states together than when considered separately.

Centralization and Co-Information. As discussed in the main text, from estimating I_L, I_G, and I_{TOT}, we derive two measures. The first is centralization, given by

$$I_{CENT} = I_G - I_L = I_{UG} - I_{UL}. \tag{8}$$

I_{CENT} is the balance of unique global versus unique local information. Such a measure could be useful for any analysis of three variables where it could be useful to know the relative dependancies between two variables and a third target variable.

The measure is commonly referred to as co-information or as the negative interaction information (3) and is given by

$$I_{CO} = I_G + I_L - I_{TOT} = I_R - I_{SYN}. \tag{9}$$

This measure is complimentary to I_{CENT} in that is looks at the balance of redundant information versus synergistic information shared between two variables and the third target variable. The higher the number, the less important it is to know both variable to learn about the target variable.

How then can we interpret a given pair of values for centralization and co-information? First, centralization gives an indication of how much unique information is present in the global state if the value is positive and in the local state if the value is negative. Then, positive co-information means that there is redundancy between the states, so knowing the less informative state becomes less important, while negative co-information means that it becomes more important to know more about both states. The two measures are together bounded according to

$$|I_{CENT}| + |I_{CO}| < I_{TOT}, \tag{10}$$

so as $|I_{CENT}|$ approaches I_{TOT}, the range of possible values for I_{CENT} shrinks and vice versa. These bounds are plotted as the solid diagonal lines that make of the diamond in the information space plotted in Fig. 6 in the main text.

C. Supplementary Note 3: Dimensionality Reduction.

Cockroach Data. The EMG signal of the cockroach, while a continuous voltage signal, is comprised of discrete spikes. These spikes can be parameterized by the number of spikes that occur within a stride and the timing that each spike occurs. The timing of spikes is normalized to the period of each stride. We first calculate the mutual information between the number of spikes and the output states, which we call count information. We then calculate the additional mutual information of the timing of the spikes given that the number of spikes is known as was done in (7).

The output states for the cockroach, as well as all time series data, is too high dimensional to be able to effectively estimate mutual information. As the data is auto-correlated with time, we expect that a low dimensional representation of the states will contain all mutual information. The simplest dimensionality reduction is to take one sample from the trajectory in phase for each stride, which we call a phase slice. We found that adding a second slice increased the estimated information significantly,

but not a third or fourth. The phases of the two slices also can result in various estimations of information as shown in Supplementary Figure 4. We thus chose two slices that were a half cycle apart that rested on the plateau of both the local and global MI landscape as shown by the black point in Supplementary Figure 4. This plateau indicates that the mutual information estimates are robust to moderate changes in the parameters of the dimensionality reduction (i.e. which particular slices are chosen), but that also there are phases in the stride that share less information than others. We confirmed that conclusions concerning centralization did not change with as the phase of these slices varied throughout the plateau region or more slices were added.

We also tried other dimensionality reduction methods such as principle component analysis (8) and partial least squares (9). We found that the two phase slice method resulted in higher estimates of mutual information than the first two components of these other methods, although overall conclusions were robust to the different methods of dimensionality reduction.

Robot Data. In the case of the robot, the control signals and the local and global states are all continuous. The control signal is the estimated axial force applied to the leg, calculated from the estimated torques of the two motors that actuate the leg using the leg Jacobian matrix (10). The local state is the extension of the leg, which is derived from the measured motor angles using the same Jacobian. The global state is the average of the leg extensions for all four legs.

We first subdivided these signals into strides by calculating a phase variable by Hilbert transforming the pitch trajectory of the robot which followed a clean oscillatory pattern as it bounded. The trajectories were normalized in time by the stride period for each stride. Then, for each experimental condition, we calculated the scores of the first principle component of the stride ensembles for each of the three variables, thus reducing the dimension of each variable to one. Using two principle components led to much less stable estimates of mutual information. Also, using the first principle component extracted more information from the data than using the phase slice method that was used to analyze the cockroach data.

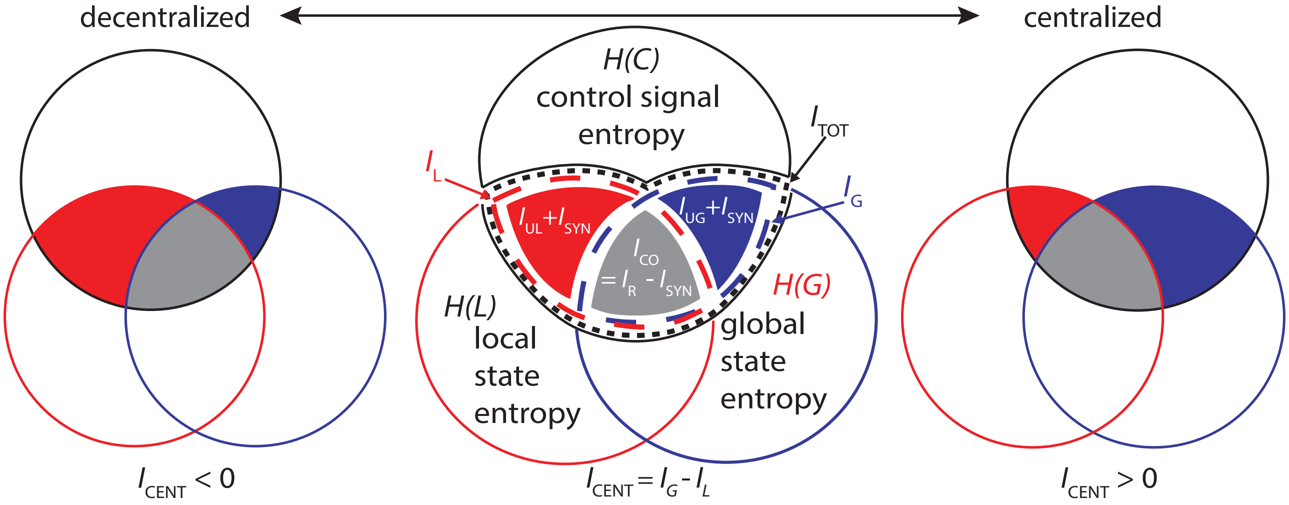

Supplementary Figure 1. Representation of overlapping entropies of the control signal, local state, and global state. We calculate I_L, the area encapsulated by the dashed red line, I_G, the area encapsulated by the dashed blue line, and I_{TOT}, which is the area encapsulated by the dotted black line. I_{CENT} is negative when there is more red than blue area, and positive when there is more blue than red area.

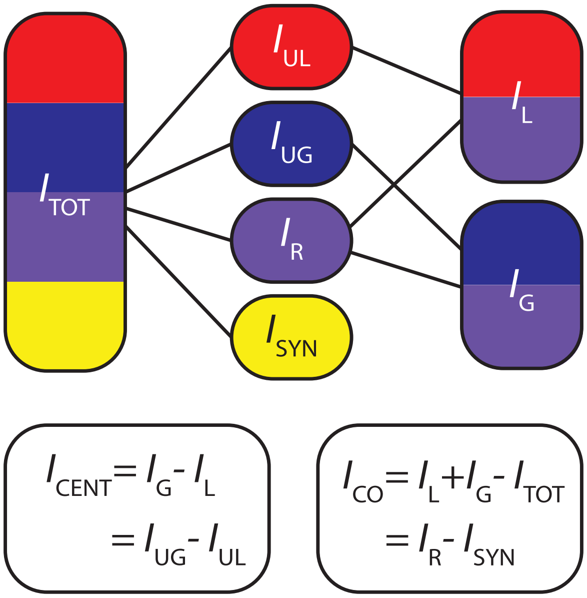

Supplementary Figure 2. The information decomposition of the trivariate case.

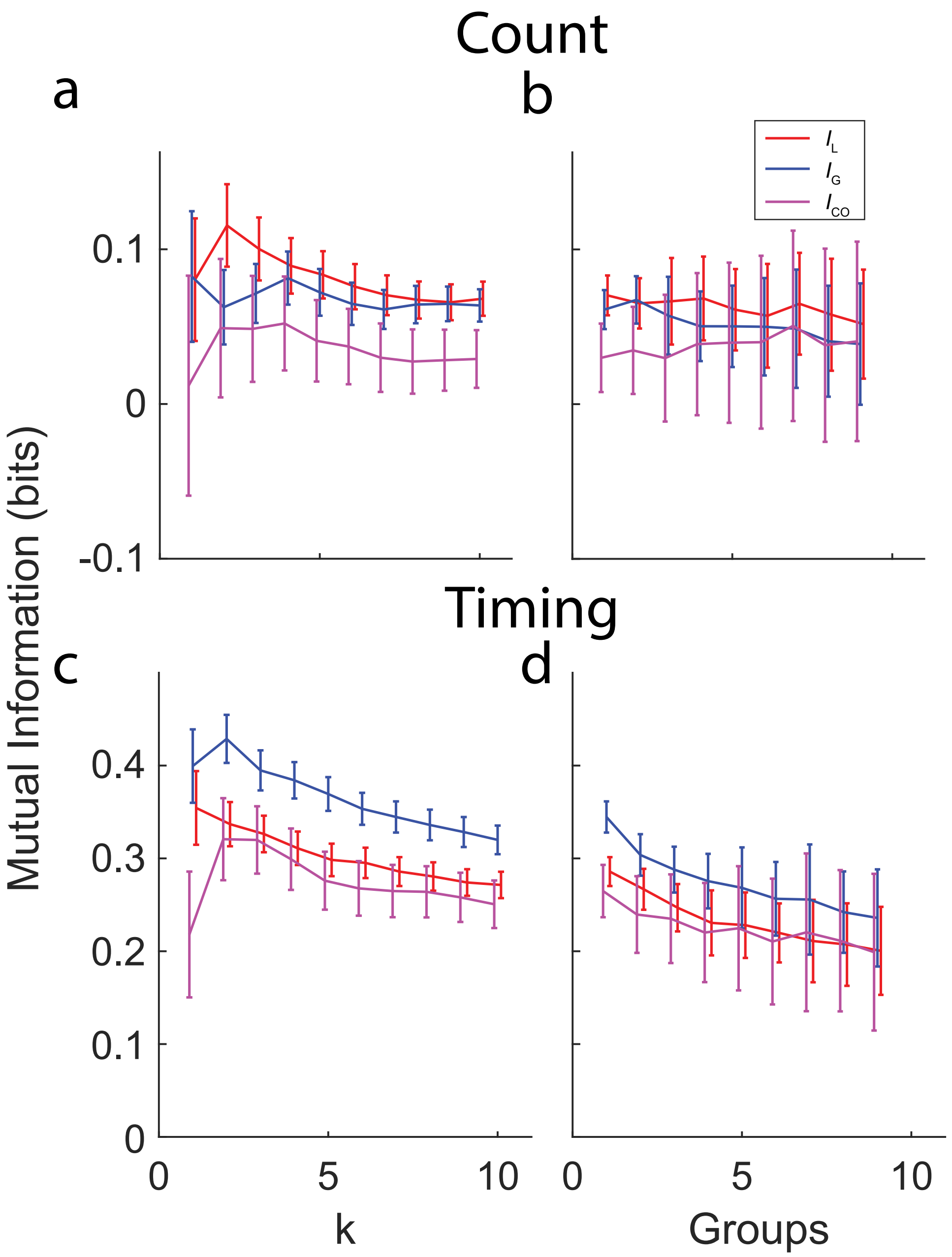

Supplementary Figure 3. Estimates of I_G and I_L while varying k and sample size. a) Information in count as k varies. b) Information in count as sample size varies by subdividing the strides into different numbers of groups. c) Information in timing as k varies. d)Information in timing as sample size varies. Error bars show the standard deviation of the estimate as calculated by the procedure given in the text and adapted from (7).

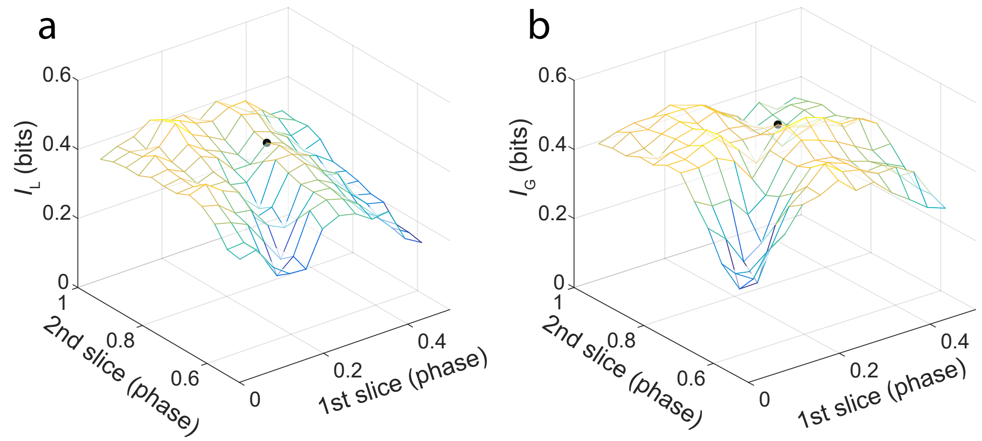

Supplementary Figure 4. Effect of phase slicing on mutual information estimates in the cockroach. a) I_L (including both count and timing) estimates depend on which two slices of the local state are considered. b) Same as (a) for the I_G. We looked for a slice pair offset by a half cycle that resulted in a estimate close to the maximum information across the ranges of parameters tested for both I_L and I_G. We selected the two slices indicated by the black point and verified that conclusions concerning centralization did not change with small variations to the phase of these slices.

References

1. Burks AW, Shannon CE, Weaver W (1951) The Mathematical Theory of Communication. *The Philosophical Review* 60(3):398.
2. Kraskov A, Stögbauer H, Grassberger P (2004) Estimating mutual information. *Physical Review E* 69(6):066138.
3. Timme N, Alford W, Flecker B, Beggs JM (2014) Synergy, redundancy, and multivariate information measures: an experimentalist's perspective. *Journal of Computational Neuroscience* 36(2):119–140.
4. Williams PL, Beer RD (2010) Nonnegative decomposition of multivariate information. *arXiv preprint arXiv:1004.2515*.
5. Ince R, Ince, A. RA (2017) Measuring Multivariate Redundant Information with Pointwise Common Change in Surprisal. *Entropy* 19(7):318.
6. Ting LH, Macpherson JM (2005) A Limited Set of Muscle Synergies for Force Control During a Postural Task. *Journal of Neurophysiology* 93(1):609–613.
7. Srivastava KH, et al. (2017) Motor control by precisely timed spike patterns. *Proceedings of the National Academy of Sciences of the United States of America* 114(5):1171–1176.
8. Pearson K (1901) Principal components analysis. *The London, Edinburgh, and Dublin Philosophical Magazine and Journal of Science* 6(2):559.
9. Sponberg S, Daniel TL, Fairhall AL (2015) Dual dimensionality reduction reveals independent encoding of motor features in a muscle synergy for insect flight control. *PLoS Computational Biology* 11(4):e1004168.
10. Kenneally G, De A, Koditschek DE (2016) Design principles for a family of direct-drive legged robots. *IEEE Robotics and Automation Letters* 1(2):900–907.
